# Screening of Panamanian Plants for Cosmetic Properties, and HPLC-Based Identification of Constituents with Antioxidant and UV-B Protecting Activities

**DOI:** 10.3797/scipharm.1409-12

**Published:** 2014-10-15

**Authors:** Niels Guldbrandsen, Maria De Mieri, Mahabir Gupta, Eleni Liakou, Harris Pratsinis, Dimitris Kletsas, Eliza Chaita, Nektarios Aligiannis, Alexios-Leandros Skaltsounis, Matthias Hamburger

**Affiliations:** 1 Division of Pharmaceutical Biology, University of Basel, Klingelbergstrasse 40, CH-4056 Basel, Switzerland; 2 CIFLORPAN, College of Pharmacy, University of Panama, Apartado 0824-00172, Panama, Republic of Panama; 3 Laboratory of Cell Proliferation & Ageing, Institute of Biosciences & Applications, NCSR “Demokritos”, 15310, Athens, Greece; 4 Korres S.A. Natural Products, 57th Athens-Lamia National Road, 32011, Inofyta, Greece; 5 Department of Pharmacognosy & Natural Products Chemistry, Faculty of Pharmacy, University of Athens, Zografou, 15771, Greece

**Keywords:** Panamanian plant extract, HPLC-based activity profiling, DPPH scavenging, UV-B protection

## Abstract

A library of 600 taxonomically diverse Panamanian plant extracts was screened for DPPH scavenging and UV-B protective activities, and the methanolic extracts of *Mosquitoxylum jamaicense, Combretum cacoucia*, and *Casearia commersionia* were submitted to HPLC-based activity profiling. The compounds located in the active time windows were isolated and identified as gallic acid derivatives and flavonoids. Gallic acid methyl ester (**3**) and digallic acid derivatives (**2, 6**) showed the highest DPPH scavenging activity (<10 μg/mL), while protocatechuic acid (**7**) and isoquercitrin (**10**) exhibited the highest UV-B protective properties.

## Introduction

The skin is the largest organ of the human body, functioning as an effective barrier against the harmful effects of the environment [1]. Several factors affect skin health and promote skin aging, such as ionizing radiation, severe physical and psychological stress, alcohol intake, poor nutrition, overeating, environmental pollution, and exposure to UV radiation. The latter is believed to contribute up to 80% of extrinsic skin damage [2].

In cosmetics, natural products play a major role as active ingredients given that they are considered by many as safer alternatives to synthetic products and, therefore, possess higher consumer acceptance. Numerous cosmetic products for dry skin, skin protection (ROS, radicals, and UV light), prevention or alleviation of skin inflammation, hyperpigmentation, and anti-aging products are commercially available [3–5].

Free radical formation can induce skin damage through a series of mechanisms leading to cell death and ultimately, to skin aging. In a search for new active ingredients for skin care products, compounds and extracts of natural origin are of significant interest [6]. The potential of purified plant compounds in skin protection is generally recognized, but plant extracts also show significant potential due to their complex composition [7].

In the field of cosmetic ingredients, relatively few studies on novel plant extracts or pure natural products have been published in recent years, and the majority of these studies were linked to ethnobotanical sources [3]. Screening of taxonomically diverse and unique plant collections is an alternative strategy to an ethnobotany-driven approach, and it has been successfully applied in the drug discovery field [8]. A diversity-oriented approach is the most successful if plants from regions of high biodiversity can be accessed. Panama is located in one of the 25 biodiversity hotspots worldwide. Despite the small surface of the country, its flora comprises 9,893 vascular plant species including 1,327 (13.4%) endemic plants [9–11]. The flora of Panama is a rich source of bioactive molecules and represents a largely untapped source for new compounds with promising activities for pharmaceutical, agrochemical, and cosmetic industries [12–14].

In an FP7 framework project aiming at the discovery of new natural products for cosmetic use, we screened a library of 600 extracts generated from a set of taxonomically diverse Panamanian plants. The focus was on the identification of plants with promising UV-protective and anti-aging properties. The best extracts were submitted to a process termed HPLC-based activity profiling [15], whereby physicochemical data recorded online are combined with bioassay data of HPLC microfractions.

A broad range of assays have been reported for the analysis of radical scavenging and antioxidant activities of natural products, and for the assessment of UV-protective properties. Free radical scavenging properties are frequently detected with the stable radical, 2,2-diphenyl-1-picrylhydrazyl (DPPH), due to the simplicity of the assay which is easily amenable to screening large numbers of samples [16]. UV protection can be readily assessed based on the capability of the test products to reverse UV-induced cell death by using the widely accepted MTT methodology [17–19].

## Results and Discussion

A library of 600 extracts prepared from Panamanian plants was screened for antioxidant capacity and the ability to protect human skin fibroblasts against UV-B-induced cell death. The screening results of the selected extracts are given in Table 1, and a flow chart for the further progression of samples is shown in Fig. 1. A total of 19 extracts were found to possess considerable radical scavenging activity, i.e. IC_50_ ≤ 30 μg/ml in the DPPH assay. These extracts were screened for their ability to protect human skin fibroblasts against UV-B-induced cytotoxicity, and three extracts were found to reduce UV-B-induced cell death to ≤15% of the control.

**Tab. 1:**
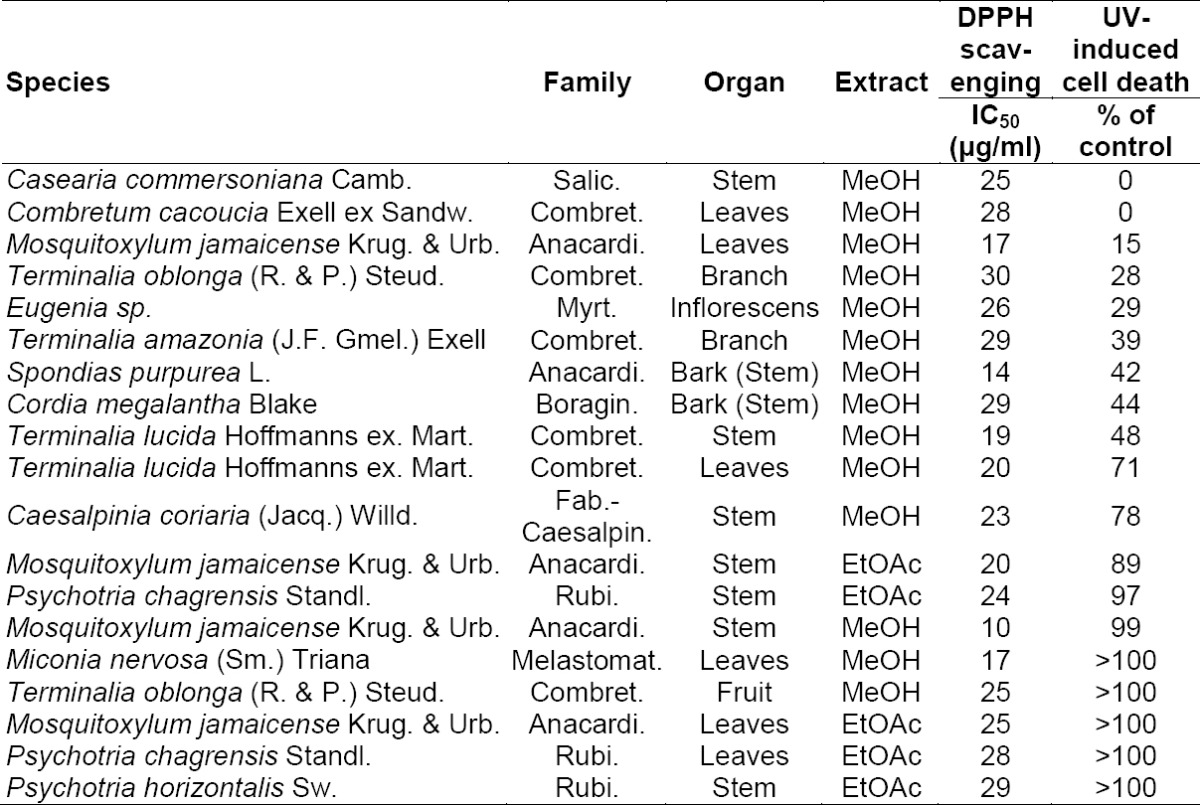
Activity data of selected extracts in DPPH and UV-B protection assays

**Fig. 1:**
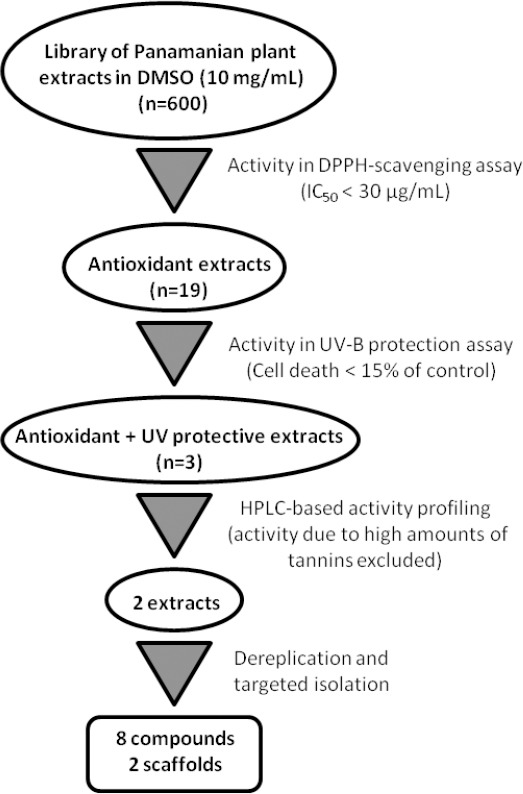
Workflow for the discovery of cosmetics from Panamanian plant extracts

Active extracts were then submitted to HPLC-based activity profiling [15] in order to track the active constituents in the extract. Time-based microfractions were collected and submitted to screens. HPLC traces and activity profiles are shown in Fig. 2. Extracts were then prioritized on the basis of HPLC traces and activity profiles. In the case of the MeOH extract of *Casearia commersionia* (Salicaceae) (Fig. 2A), a broad window of activity corresponded to a broad hump in the baseline of the HPLC chromatogram. This was a strong indicator for the presence of tannins, and the extract was therefore excluded from the follow-up. In contrast, for the MeOH extracts of *Mosquitoxylum jamaicense* (Anacardiaceae) (Fig. 2B) and *Combretum cacoucia* (Combretaceae) (Fig. 2C), the activity profile correlated with discrete peaks in the chromatograms, even though broad humps in the baseline were also indicative of tannins. These two extracts were selected for characterization of the active constituents.

**Fig. 2:**
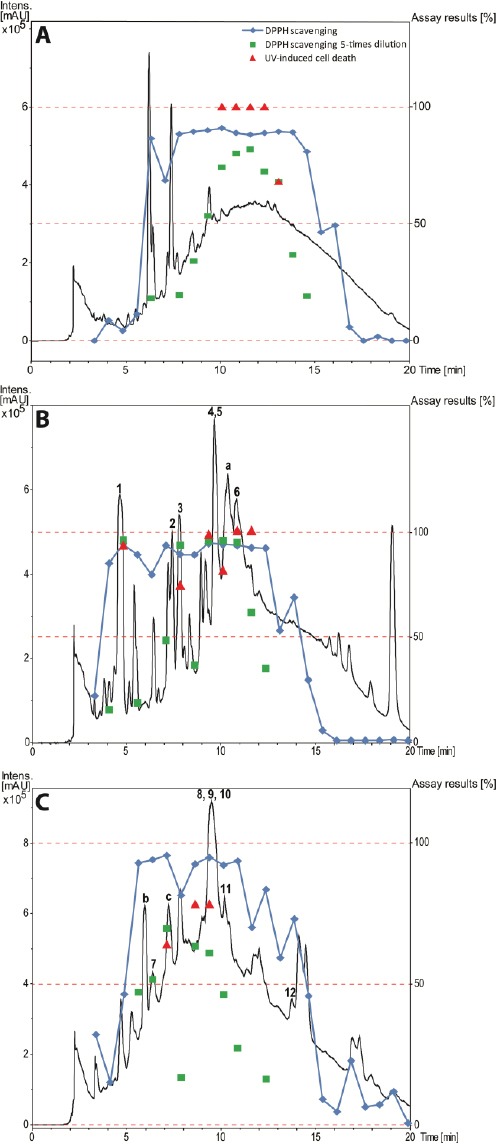
HPLC-based activity profiling of the three selected plant extracts in the active time window (0–20 min). SunFire C_18_ column (150 × 10 mm i.d., 5 μm); 5–100% MeCN/0.1% aqueous formic acid for 30 min and 100% MeCN/0.1% aqueous formic acid for 5 min, 4 mL/min; time-based fractionation; detection: 200–500 nm, maxplot. **A** *Casearia commersoniana* (stems) MeOH extract. **B** *Mosquitoxylum jamaicense* (leaves) MeOH extract. **C** *Combretum cacoucia* (leaves) MeOH extract. The assay results are expressed as the radical scavenging capacity of the microfractions in the DPPH assay, compared to gallic acid as the positive control, and as cell death in the UV-B protection assay, as compared to the UV-B irradiated cells without the addition of fractions.

The tannins in the two extracts were removed by filtration over polyamide (Figs 1S and 2S, Supporting Information). The MeOH leaf extract of *Mosquitoxylum jamaicense* showed activity in time windows corresponding to UV-absorbing peaks in the HPLC chromatogram (Fig. 2B). The tannin-depleted fractions from polyamide (Fig 1S, Fig 1 Supporting Information) were submitted to further purification by HPLC. Peak **1** was identified as gallic acid (Fig. 3), by spiking with a commercial reference and by NMR spectroscopy. Given that the radical scavenging and antioxidant properties of gallic acid are known [20], the compound was not pursued further. The other two early-eluting peaks were identified as a 7:3-mixture of meta- and para-digallic acid (**2**) [21] and a gallic acid methyl ester (**3**) [22]. Both compounds were found to possess good radical scavenging activity (Table 2), which was in accordance with the well-known radical scavenging properties of gallic acid [20]. In addition, compounds **2** and **3** showed protective capacity against UV-B radiation.

**Fig. 1S:**
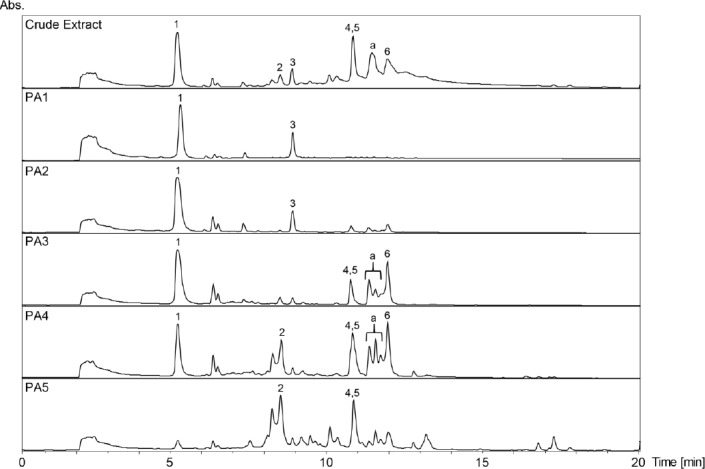
HPLC-DAD chromatograms of the crude extract and its polyamide fractions (PA1-PA5) of *Mosquitoxylum jamaicense*. SunFire C18 column (150 × 3 mm i.d., 3.5 μm); 5–100% MeCN/0.1% aqeous formic acid in 30min, 0.4 mL/min; detection: 210–700nm, maxplot.

**Fig. 2S:**
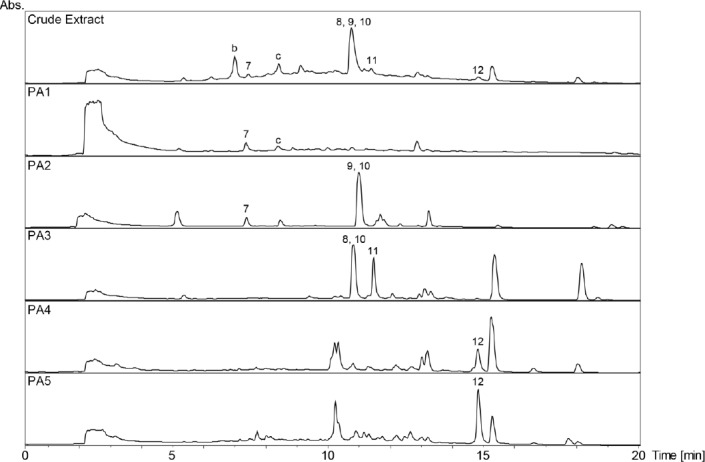
HPLC-DAD chromatograms of the crude extract and its polyamide fractions (PA1-PA5) of *Combretum cacoucia*. SunFire C18 column (150 × 3 mm i.d., 3.5 μm); 5–100% MeCN/0.1% aqeous formic acid in 30min, 0.4 mL/min; detection: 210–700nm, maxplot.

**Fig. 3:**
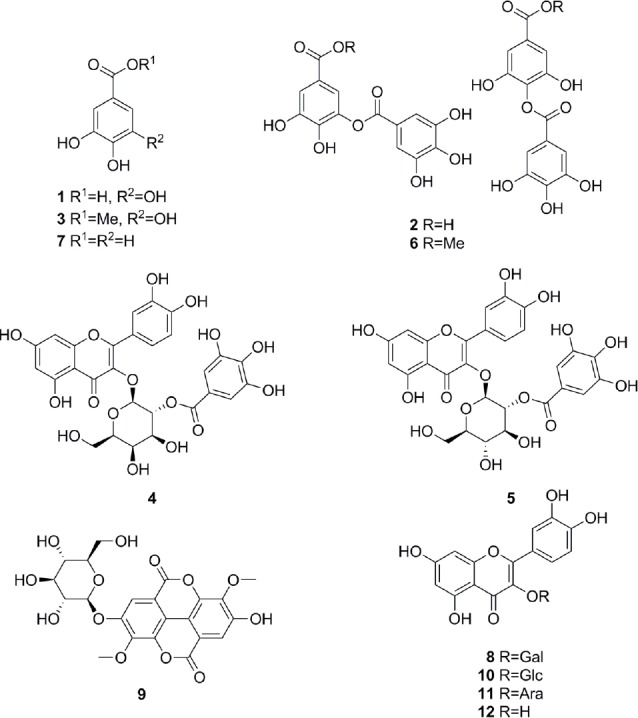
Structures of identified compounds, gallic acid (1), 7:3-mixture of meta- and para-digallic acid (2), gallic acid methyl ester (3), quercetin-3-*O*-(2’’-*O*-galloyl)-β-galactopyranoside (4), quercetin-3-*O*-(2’’-*O*-galloyl)-β-glucopyranoside (5), 7:3-mixture of meta- and para-digallic acid methyl ester (6), protocatechuic acid (7), hyperoside (8), 3,3’-dimethylellagic acid 4-*O*-β-glucopyranoside (9), isoquercitrin (10), guaijaverin (11), and quercetin (12)

**Tab. 2:**
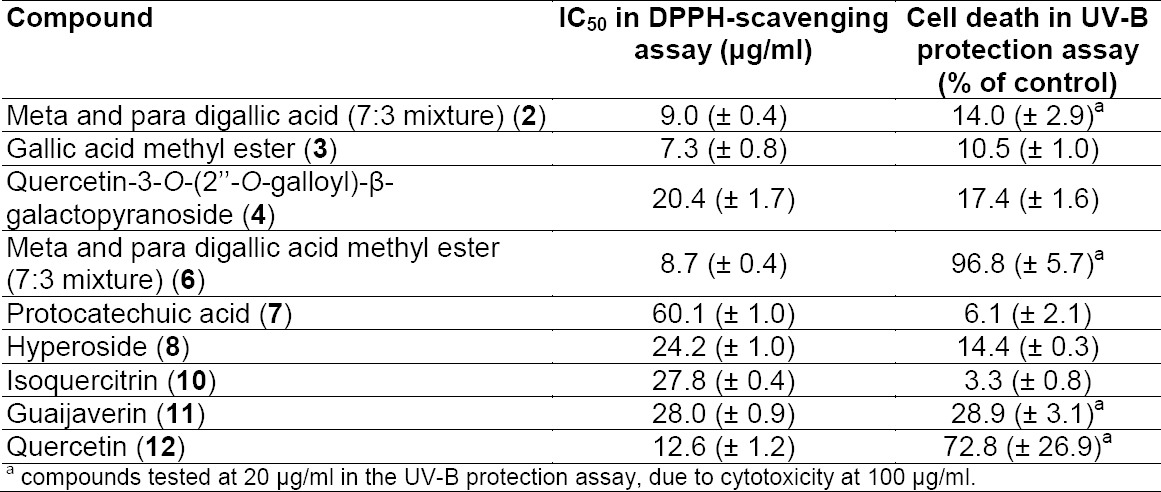
Activity data of pure compounds

The major peak at t_R_ 10 min in the HPLC chromatogram consisted of two co-eluting flavonol glycosides **4** and **5**. Compound **4** was purified and identified as quercetin-3-*O*-(2’’-O-galloyl)-β-galactopyranoside [23], while quercetin-3-O-(2’’-O-galloyl)-β-glucopyranoside (**5**) [24] was identified from a fraction containing **4** and **5**. Compound **4** was found to be a slightly weaker antioxidant and photoprotectant than **2** and **3** (Table 2). Peak **a** consisted of several co-eluting compounds and was not pursued further. Peak **6** was enriched by filtration over polyamide, and HPLC purification afforded a 7:3-mixture of meta- and para-digallic acid methyl ester (**6**) [25]. Compound **6** showed radical scavenging activity comparable to **2** and **3,** but no protective capacity against UV-B (Table 2).

The activity profile recorded for the methanolic leaf extract of *Combretum cacoucia* showed a zone of radical scavenging capacity between t_R_ 5 and 15 min (Fig. 2C). Filtration over polyamide afforded five tannin-depleted fractions (Fig. 2S, Fig. 2 Supporting Information) from which peaks **b** and **c** had disappeared. The main peak at t_R_ 10 min consisted of three compounds which were further purified and identified as hyperoside (**8**) [26], 3,3’-dimethylellagic acid 4-*O*-β-glucopyranoside (**9**) [27], and isoquercitrin (**10**) [26]. Compound **9** could not be obtained at a purity of ≥95% required for further testing. Compounds **8** and **10** were found to possess DPPH scavenging activity and the capacity to protect human skin fibroblasts from UV-B radiation (Table 2). Isoquercitrin (**10**) almost completely blocked cell death. Three minor peaks (**7**, **11**, and **12**) in the active time window were enriched in fraction PA2, and were identified as protocatechuic acid (**7**) [28], guaijaverin (**11**) [29], and quercetin (**12**) [30]. Compound **7** could only be obtained at 90% purity, with traces of phenolic glycosides as contaminants. The compound was nevertheless tested and exhibited weak antioxidant activity, but very good UV-B protection. Quercetin (**12**) and its glycosides **8**, **10**, and **11** showed significant free radical scavenging properties as previously reported [31–33], but only the glycosides **8**, **10** and **11** showed significant UV-protective activity (Table 2). However, the lower test concentrations for compounds **11** and **12** had to be taken into account.

The screening of a taxonomically diverse library of Panamanian plant extracts followed by an activity-driven identification of radical scavenging and UV-B protecting properties led to the identification of a series of known polyphenols. The example shows that the profiling approach can be efficiently used not only for the discovery of bioactive compounds of pharmaceutical, but also of cosmetic interest.

## Experimental

### Chemicals and General Experimental Procedures

Quercetin (**12**, >98%) was purchased from Sigma-Aldrich. Gallic acid (**1**, >98%), hyperoside (**8**, >95%), isoquercitrin (**10**, >95%), and polyamide (particle size: 0.05–0.16 mm) were from Carl Roth. HPLC-grade acetonitrile and methanol (Reuss Chemie AG), and distilled water were used for HPLC separations.

Preparative HPLC was carried out on an LC 8A preparative liquid chromatograph equipped with a SPD-M10A VP PDA detector (all Shimadzu). A SunFire C_18_ column (150 × 30 mm i.d., 5 μm; Waters) connected to a pre-column (10 × 10 mm) was used, at a flow rate of 20 mL/min. HPLC-based activity profiling was performed on an Agilent 1100 system equipped with a PDA detector. A SunFire C_18_ column (150 × 10 mm i.d., 5 μm; Waters) connected to a pre-column (10 × 10 mm) was used. The flow rate was 4 mL/min. Time-based fractions were collected with a Gilson FC204 fraction collector. ESI-MS spectra were obtained on an Esquire 3000 Plus ion trap mass spectrometer (Bruker Daltonics). NMR spectra were recorded on an Avance III 500 MHz spectrometer (Bruker BioSpin) equipped with a 1-mm TXI microprobe.

### Plant Material

The leaves of *Mosquitoxylum jamaicense* were collected in May 2000 in Parque Nacional Soberanía, Camino del Oleoducto, Km 17, Panama. The leaves of *Combretum cacoucia* were collected in April 1995 in Costa Arriba, San Antonio, Colón, Panama. *Casearia commersoniana* was collected in Peninsula Gigante, Chorrera in June, 1995. The plant material was identified by Alex Espinosa and voucher specimens have been deposited at the Herbarium of the University of Panama (PMA). Also, vouchers were kept at the Division of Pharmaceutical Biology, University of Basel: Nr. 857 (*M. jamaicense*), Nr. 859 (*C. cacoucia*), and 903 (*C. commersoniana*).

### HPLC-Based Activity Profiling

Extract solutions dissolved in DMSO (50 mg/mL) were separated by semi-preparative HPLC. Two aliquots of 200 μL corresponding to 10 mg of the extract were injected. A gradient of 5–100% MeCN in 30 min in 0.1% aqueous formic acid, followed by 100% MeCN over 5 min was used. Fractions of 0.75 min were collected from t = 3 min to t = 33 min. Fractions were transferred into 96-deepwell plates, evaporated, and submitted to screening.

### Extraction and Isolation

Powdered leaves of *M. jamaicense* (704.6 g) were percolated with 12 L MeOH to afford 198.5 g of the extract. A portion (20.2 g) of the extract was re-dissolved in 200 mL MeOH and submitted to filtration over a polyamide (200 g) column. Four fractions (PA1-PA4) of 500 mL each, and one fraction (PA5) of 3 L were collected. A portion (1.01 g) of PA1 (4.99 g) was separated by preparative HPLC (16% MeCN in 0.1% aqueous formic acid) to afford gallic acid methyl ester (**3**, 14.3 mg, t_R_ 11.3 min). Preparative HPLC of fraction PA3 (338.9 mg) (50–80% MeOH in 0.1% aqueous formic acid over 15 min) yielded a 7:3-mixture of meta- and para-digallic acid methyl ester (**6**, 24.5 mg, t_R_ 12.5 min). Fraction PA5 (629.2 mg) was separated on a Sephadex LH-20 column (5 × 75 cm i.d.) and eluted with MeOH to give 17 fractions (Fr. 1-17). Preparative HPLC (25% aqueous MeOH with 0.1% formic acid) of Fr. 11 (136.3 mg) afforded a 7:3-mixture of meta- and para-digallic acid (**2**, 67.8 mg, t_R_ 12.1 min). From Fr. 13 (69.8 mg), quercetin-3-*O*-(2’’-*O*-galloyl)-β-galactopyranoside (**4**, 8.2 mg, t_R_ 11.3 min) and a mixture of quercetin-3-*O*-(2’’-*O*-galloyl)-β-glucopyranoside (**5**, t_R_ 11.5 min) and **4** were obtained by preparative HPLC (44% aqueous MeOH with 0.1% formic acid).

Powdered leaves of *C. cacoucia* (125.2 g) were percolated with MeOH (3 L) to afford 14.1 g of the extract. A portion (10.1 g) of the extract was re-dissolved in 200 mL MeOH and filtered over a polyamide (200 g) column. Two fractions (PA1-PA2) of 500 mL each, two fractions (PA3-PA4) of 1 L each, and one fraction (PA5) of 3 L were collected. Polyamide fractions were submitted to preparative HPLC. A portion (1.07 g) of fraction PA1 (3.79 g) was separated with a gradient of MeCN in 0.05% aqueous formic acid (5–40% over 15 min) to afford protocatechuic acid (**7**, 8.5 mg, t_R_ 10.1 min). From PA2 (473.4 mg), 3,3’-dimethylellagic acid 4-*O*-β-glucopyranoside (**9**, 7.8 mg, t_R_ 6.8 min) and isoquercitrin (**10**, 20.2 mg, t_R_ 7.8 min) were isolated using 50% MeOH in 0.1% aqueous formic acid. Final purification of **10** was with 20% MeCN in 0.1% aqueous formic acid (1.6 mg, t_R_ 18.6 min). Fraction PA3 (283.8 mg) was separated with a gradient of MeCN in 0.05% aqueous formic acid (20–60%, 20 min) to afford a mixture of hyperoside (**8**) and isoquercitrin (**10**) (35.5 mg, t_R_ 7.9 min), and guaijaverin (**11**, 14.9 mg, t_R_ 8.9 min). Quercetin (**12**, 16.7 mg, t_R_ 11.1 min) was isolated from fraction PA4 (161.8 mg) using a gradient of 30-60% MeCN in 0.05% aqueous formic acid over 20 min.

Compounds were identified with the aid of ^1^H- and 2D-NMR, and ESI-MS spectroscopy, and by comparison with the literature data. The purity of the isolated compounds was >95% as determined by NMR except for compounds **7** (90%) and **9** (<70%).

### DPPH Radical Scavenging Assay

The antioxidant potential of the test samples was monitored by the change in optical density of the DPPH radical. A stock solution of 0.314 mM DPPH in EtOH was prepared. This stock solution was prepared fresh every day. Extracts were initially tested at 200 μg/ml. Samples that exhibited a strong DPPH scavenging activity, i.e > 80% scavenging, were further evaluated at lower concentrations.

Dry microfractions of the selected extracts in 96-deepwell plates were dissolved in DMSO and tested directly against DPPH scavenging. When a large number of active microfractions appeared for one extract, the most active fractions were tested at a 5-fold dilution. In a 96-well plate, 10 μl of the sample (extract/ fraction/ compound) in DMSO and 190 μl of DPPH solution were mixed and incubated in the dark for 30 min at ambient temperature. Absorbance was measured at 517 nm using an Infinite M200Pro plate reader (Tecan, Männedorf, Switzerland). Measurements were done in triplicate. Blanks for every sample without DPPH were also measured. Gallic acid was used as the positive control. The percentage of DPPH scavenging was estimated by the following equation:

{[(A-B)-(C-D)]/(A-B)}x100, where A: Control (without sample), B: Blank (without sample, without DPPH), C: Sample, D: Blank sample (without DPPH). IC_50_ values, which are defined as the amount of sample necessary to decrease the initial free radical concentration by 50%, were estimated for the isolated compounds and most active extracts.

### Cell Protection Against UV-B Irradiation

A human skin fibroblast cell line (AG01523; Coriell Institute for Medical Research, Camden, NJ, USA) was used for the assessment. Cells were routinely cultured in Dulbecco’s Modified Eagle Medium (DMEM) supplemented with antibiotics (100 IU/ml penicillin; 100 μg/ml streptomycin) and 15% Fetal Bovine Serum (FBS) in an environment of 5% CO_2_, 85% humidity, at 37°C, and subcultured once a week at a 1:2 split ratio, using a trypsin–citrate solution (0.25%–0.3%, respectively). Cell counting after trypsinization was performed using a Coulter counter.

For assessing the possible cytotoxicity of the samples (extracts, fractions, or isolated compounds), cells were plated in flat-bottom, tissue culture-treated 96-well plates at a density of 5,000 cells/well. After 48 hours of growth, the medium was changed to serum-free, phenol red-free DMEM, and serial dilutions of the test samples were added. The corresponding dilutions of dimethylsulfoxide (DMSO) served as negative controls. Following incubation with the test samples for 72 hours, the medium was changed to serum-free, phenol red-free DMEM containing 1 mg/ml 3-(4,5-dimethylthiazol-2-yl)-2,5-diphenyltetrazolium bromide (MTT) as described by Kostakis et al. [34]. After incubation with MTT for 4 hours, the medium was discarded, and the MTT-formazan crystals were dissolved in isopropanol. Absorbance was measured at 550 nm (reference wavelength; 690 nm) in an Infinite M200 microplate reader (Tecan) using Magellan^™^ software.

The highest non-cytotoxic concentration of each sample (extract, fraction, or isolated compound) was tested for the ability to protect human skin fibroblasts against toxicity of UV-B irradiation. Cells were plated in 96-well-plates and left to grow as described above. Then samples were added at the test concentrations determined as described above, along with serum-free, phenol red-free DMEM. After incubation for 18 hours, cells were subjected to UV-B irradiation for 10 min (corresponding to 726 mJ/cm^2^) using a black box equipped with a closely spaced array of four Sankyo Denki UV-B lamps (Zhe Jiang, China) emitting between 280 nm and 360 nm (peak at 306 nm). Following further incubation for 72 hours, cytotoxicity was estimated using the MTT-method, as described in the previous paragraph. The plates treated in an identical manner, except for the UV-B irradiation, were used as the controls. The UV-B-protective capacity of the samples was calculated using the following equation: % Cell death = [1-(D/C)/1-(B/A)]x100 where A = absorbance (DMSO untreated), B = absorbance (DMSO UV-B-treated), C = absorbance (test sample untreated), and D = absorbance (test sample UV-B-treated). A value of 100 indicated the absence of protection, and 0 indicated the maximum protective capacity against UV-B.

**Tab. 1S:**
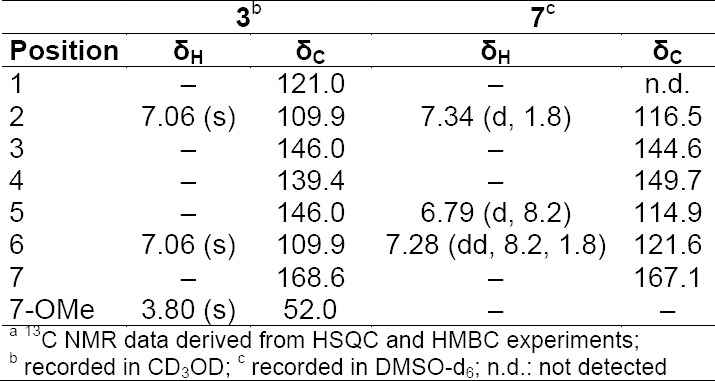
^1^H and ^13^C NMR^a^ data (500 MHz) of compounds **3** and **7**

**Tab. 2S:**
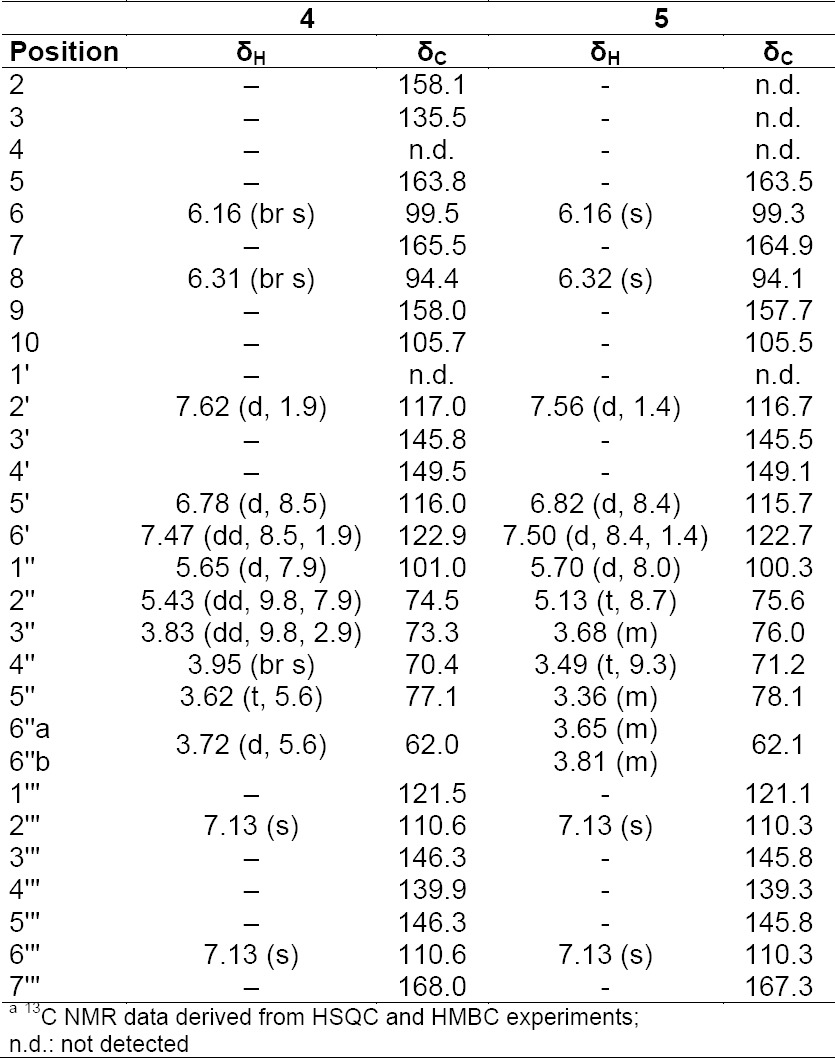
^1^H and ^13^C NMR^a^ data (500 MHz) of compounds **4** and **5** in CD_3_OD

**Tab. 3S:**
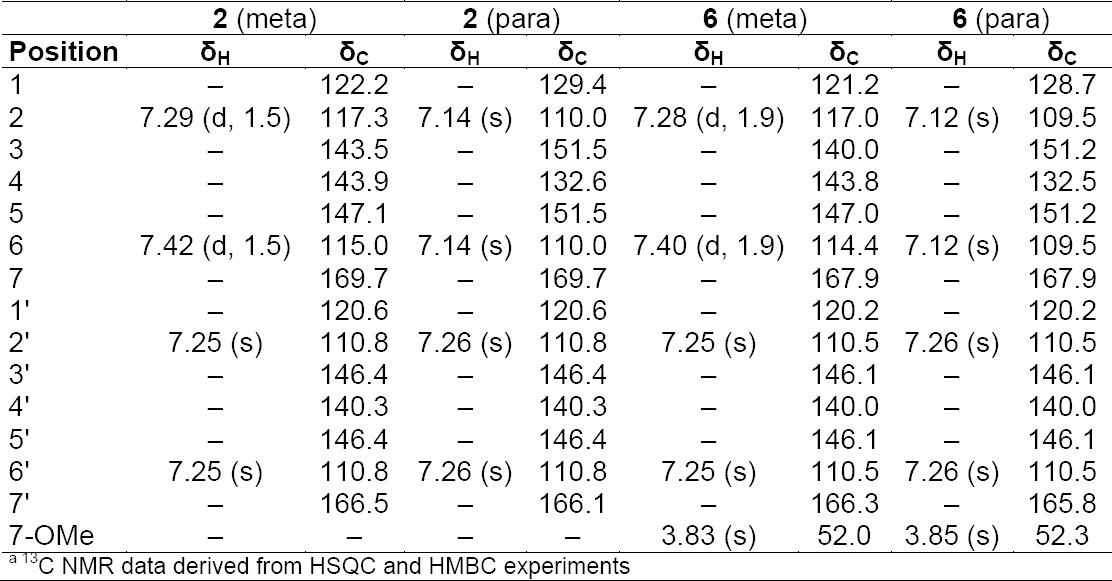
^1^H and ^13^C NMRa data (500 MHz) of compounds **2** and **6** in CD3OD

**Tab. 4S:**
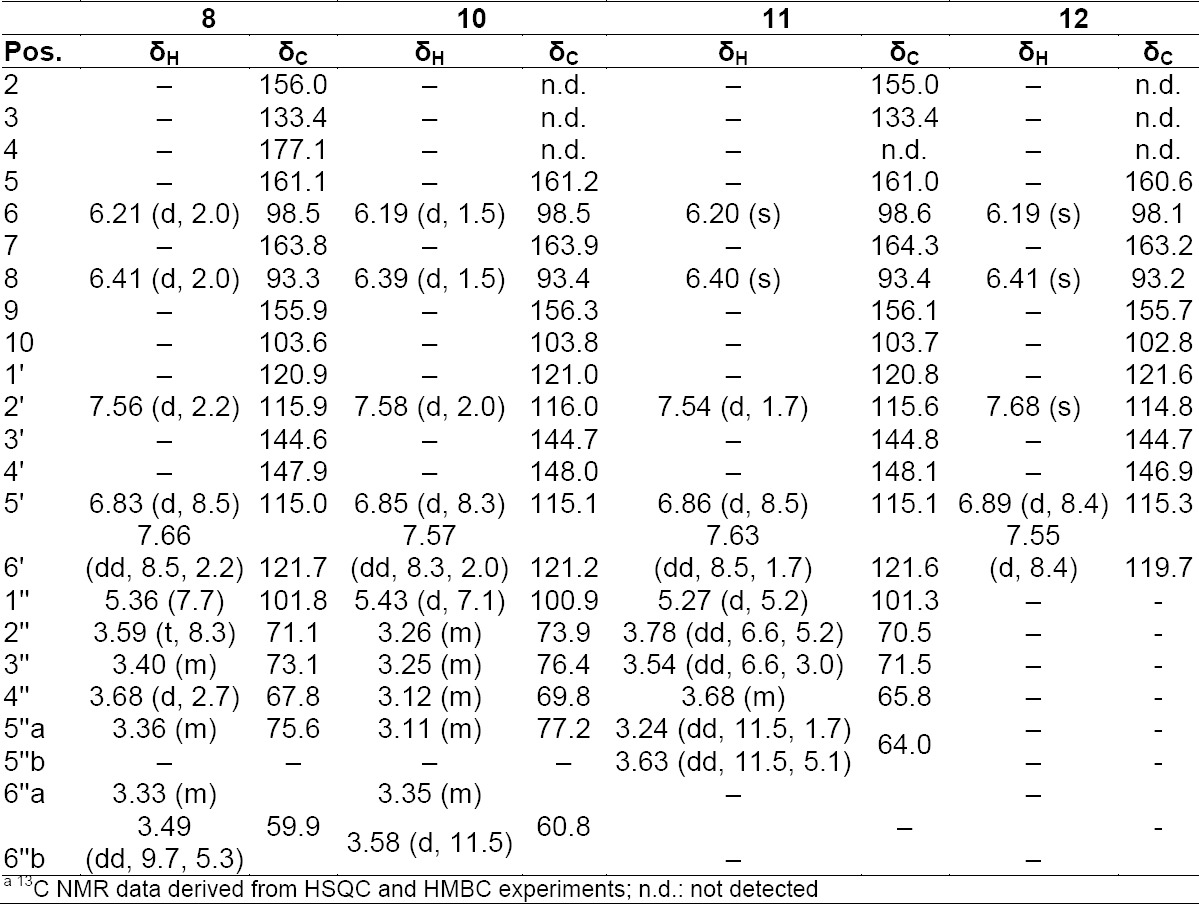
^1^H and ^13^C NMR^a^ data (500 MHz) of compounds **8, 10, 11**, and **12** in DMSO-d_6_

**Tab. 5S:**
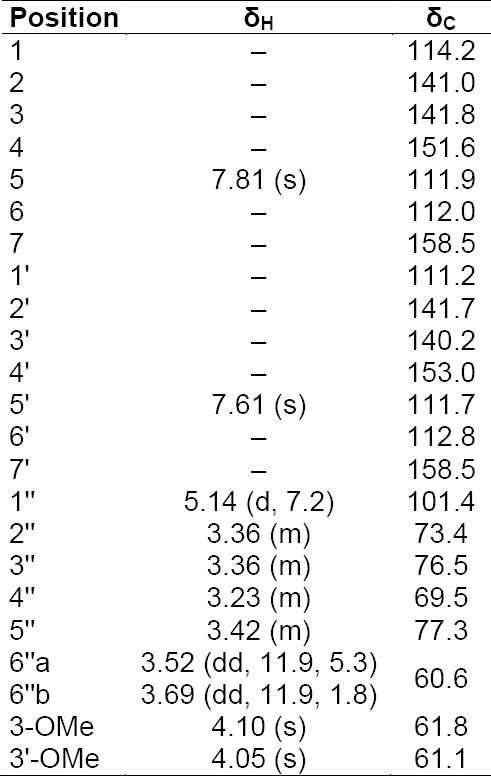
^1^H and ^13^C NMR data (500 MHz) of compound **9** in DMSO-d_6_

## Supporting Information

The HPLC profiles of the polyamide fractions and NMR data of compounds **2**–**12** are available in the online version (Type: PDF, Size: ca. 0.3 MB): http://dx.doi.org/10.3797/scipharm.1409-12
